# Heat shock factor HSFA2 fine-tunes resetting of thermomemory via plastidic metalloprotease FtsH6

**DOI:** 10.1093/jxb/erac257

**Published:** 2022-06-15

**Authors:** Mastoureh Sedaghatmehr, Benno Stüwe, Bernd Mueller-Roeber, Salma Balazadeh

**Affiliations:** Max Planck Institute of Molecular Plant Physiology, Am Mühlenberg, Potsdam-Golm, Germany; Max Planck Institute of Molecular Plant Physiology, Am Mühlenberg, Potsdam-Golm, Germany; Max Planck Institute of Molecular Plant Physiology, Am Mühlenberg, Potsdam-Golm, Germany; Institute of Biochemistry and Biology, University of Potsdam, Karl-Liebknecht-Straße, Haus, Potsdam-Golm, Germany; Center of Plant Systems Biology and Biotechnology (CPSBB), Plovdiv, Bulgaria; Max Planck Institute of Molecular Plant Physiology, Am Mühlenberg, Potsdam-Golm, Germany; University of Glasgow, UK

**Keywords:** *Arabidopsis thaliana*, FtsH6, heat stress, HSFA2, HSP21, thermomemory, thermorecovery

## Abstract

Plants ‘memorize’ stressful events and protect themselves from future, often more severe, stresses. To maximize growth after stress, plants ‘reset’ or ‘forget’ memories of stressful situations, which requires an intricate balance between stress memory formation and the degree of forgetfulness. *HEAT SHOCK PROTEIN 21* (*HSP21*) encodes a small heat shock protein in plastids of *Arabidopsis thaliana*. HSP21 functions as a key component of thermomemory, which requires a sustained elevated level of HSP21 during recovery from heat stress. A heat-induced metalloprotease, filamentation temperature-sensitive H6 (FtsH6), degrades HSP21 to its pre-stress abundance, thereby resetting memory during the recovery phase. The transcription factor heat shock factor A2 (HSFA2) activates downstream genes essential for mounting thermomemory, acting as a positive regulator in the process. Here, using a yeast one-hybrid screen, we identify HSFA2 as an upstream transactivator of the resetting element FtsH6. Constitutive and inducible overexpression of *HSFA2* increases expression of *FtsH6*, whereas it is drastically reduced in the *hsfa2* knockout mutant. Chromatin immunoprecipitation reveals *in planta* binding of HSFA2 to the *FtsH6* promoter. Importantly, overexpression of *HSFA2* improves thermomemory more profoundly in *ftsh6* than wild-type plants. Thus, by activating both memory-supporting and memory-resetting genes, HSFA2 acts as a cellular homeostasis factor during thermomemory.

## Teaser

The transcription factor HSFA2 fine-tunes a balance between prolongation and resetting of thermomemory in Arabidopsis via the regulation of both memory-supporting and memory-resetting genes.

## Introduction

Abiotic stress impairs the growth, development, and productivity of plants. An inherent ability to tolerate certain levels of stress, called basal tolerance, enables plants to combat acute stress. However, in nature, abiotic stresses are often repetitive and diverse. During their evolution, plants have, therefore, established a particular molecular and physiological inventory allowing them to survive and be reproductive under conditions of recursive and multiple stresses (Bruce *et al.*, 2007; Hilker *et al.*, 2016). A moderate and non-lethal abiotic stress is called a priming stress; it elicits a cellular machinery that allows plants to faithfully evade a later-arriving, often more severe, so-called triggering stress (Hilker *et al.*, 2016).

Most of the cellular changes induced by a priming stress are rapidly reversed to the pre-stress condition when the stress fades, allowing plants to ‘forget’ stressful situations and relocate available resources towards growth and reproduction in the following ‘stress-free’ period of their existence. Notably, however, some of the priming-triggered changes remain for an extended period of time after disappearance of the stress, thereby manifesting a ‘stress memory’ that helps plants to more effectively combat a later-arriving stress (Bruce *et al.*, 2007; Hilker *et al.*, 2016; Balazadeh, 2022). Memory and forgetfulness represent opposing, but interrelated, objectives with specific costs and benefits. Appropriately balancing the processes underlying stress memory and the extent of forgetfulness (resetting) is required for the successful development, growth, and reproduction of plants in fluctuating natural environments (Crisp *et al*., 2016, 2017; Zhang *et al.*, 2020).

A rise in environmental temperature above the optimum (e.g. 20–28 °C) poses a critical threat to plants termed heat stress (HS), which often occurs in the range of 37–45 °C. HS leads to misfolding and denaturation of proteins, which can severely impair the integrity of the cellular proteome if not curbed by protective mechanisms. To cope with HS, sessile plants have evolved transcriptional networks governed by heat shock factors (HSFs), which are often more numerous than those in other higher eukaryotes. In Arabidopsis and soybean, for example, 21 and 52 genes encoding HSFs have been identified, respectively, while the presence of fewer than 10 *HSF* genes is typical in animals (Nover *et al.*, 2001; Scharf *et al.*, 2012). In Arabidopsis, HSFs are grouped into classes A, B, and C, based on structural features. There are 15 members in class A, five members in class B, and one member in class C (Baniwal *et al.*, 2004). HSFA1s directly induce the expression of genes encoding HS-responsive transcription factors such as *MULTIPROTEIN BRIDGING FACTOR 1C* (*MBF1C*), *DEHYDRATION-RESPONSIVE ELEMENT BINDING PROTEIN 2A* (*DREB2A*), *HEAT SHOCK FACTOR A2* (*HSFA2*), *HSFA7s*, *HSFBs*, and others. These HSFA1s-induced transcription factors regulate the expression of a cascade of HS-induced transcription factors and other genes (Guo *et al.*, 2016; Ohama *et al.*, 2017).

HSFA2 is a key positive regulator of thermomemory in Arabidopsis (Charng *et al.*, 2007; Lämke *et al.*, 2016; Liu *et al.*, 2019; Friedrich *et al.*, 2021). The expression of *HSFA2* is highly induced by HSFA1s following heat exposure (Nishizawa-Yokoi *et al.*, 2011) and a splice variant, called *HSFA2-*Ⅲ, is involved in the self-regulation of *HSFA2* expression after high temperature (Liu *et al.*, 2011). The *hsfa2* knockout mutant shows defective thermomemory (Charng *et al.*, 2007) and, in accordance with this, the expression of HS-induced genes is strongly reduced. Several downstream targets of HSFA2, including genes encoding different heat shock proteins (e.g., *HSP18*, *HSP21*, *HSP22*), *HEAT-STRESS-ASSOCIATED 32-KD PROTEIN* (*HSA32*), *ASCORBATE PEROXIDASE2* (*APX2*), and *FRUCTOSE-BISPHOSPHATE ALDOLASE 6* (*FBA6*) (Nishizawa *et al.*, 2006; Schramm *et al.*, 2006; Charng *et al.*, 2007; Lämke *et al.*, 2016; Olas *et al.*, 2021), have been identified. HSP21, HSA32, and FBA6 are crucial components of thermomemory in plants (Charng *et al.*, 2006; Wu *et al.*, 2013; Sedaghatmehr *et al.*, 2016; Olas *et al.*, 2021).

Proteases are enzymes that mediate protein degradation by cleaving peptide bonds. They play important roles in controlling and maintaining cellular functions (Schaller, 2004) and are encoded by hundreds of genes in plants (van der Hoorn, 2008). The expression and activity of proteases are regulated in response to developmental and environmental cues (Sinvany-Villalobo *et al.*, 2004; Zaltsman *et al.*, 2005; Roberts *et al.*, 2012). Filamentation temperature-sensitive H6 (FtsH6) belongs to the group of metalloproteases. We previously reported that FtsH6 resets HSP21 abundance to its pre-stress condition during the thermorecovery phase. We observed that the decrease in HSP21 abundance during the thermorecovery phase was retarded in *ftsh6* loss-of-function mutants, and these plants displayed enhanced thermomemory compared with wild-type (WT) plants (Sedaghatmehr *et al.*, 2016). It was previously shown that the expression of *Ftsh6* is highly induced by heat treatment in Arabidopsis and other plant species including tomato, wheat, sorghum, and rapeseed, whereas it is not expressed in plants grown at normal conditions (Sun *et al.*, 2006; Johnson *et al.*, 2014; Yu *et al.*, 2014; Xue *et al.*, 2015). However, transcription factors regulating the expression of *FtsH6* during HS have, to our knowledge, not been reported yet.

In the present study, we discovered that HSFA2 activates the expression of the negative regulator of thermomemory, *FtsH6*, by interacting with its promoter, which contains multiple HSFA2 binding sites. Moreover, we show that thermopriming-enhanced expression of *FtsH6* is reduced in the *hsfa2* mutant but enhanced in transgenic plants overexpressing *HSFA2* from either constitutive or chemically inducible promoters. In accordance with this, the overexpression of *HSFA2* improved thermomemory more profoundly in *ftsh6-* than Col-0-transformed plants. Our results thus identify HSFA2 as a homeostatic control factor during thermomemory.

## Materials and methods

### Plant materials, growth conditions, and HS treatments

*Arabidopsis thaliana* (L.) Heynh. accession Columbia (Col-0) was used as the WT. The mutants *hsfa2* and *ftsh6*, as well as the transgenic line *HSFA2prom:HSFA2-YFP*/*hsfa2*, were previously described (Stief *et al.*, 2014; Lämke *et al.*, 2016; Sedaghatmehr *et al.*, 2016). Seeds were germinated on 0.5× Murashige and Skoog agar medium supplemented with 1% (w/v) sucrose. Seedlings were grown under a diurnal cycle of 16 h light (120 μmol m^−2^ s^−1^) at 22 °C, and 8 h dark at 22 °C. HS treatments were previously described (Stief *et al.*, 2014; Sedaghatmehr *et al.*, 2016). Briefly, 5-day-old Arabidopsis seedlings were subjected to a heat regime of 37 °C for 1.5 h, followed by recovery at 22 °C for 1.5 h and then a further heat treatment of 44 °C for 45 min; thereafter, plants were returned to normal growth conditions (thermorecovery phase) and analysed. To assess the thermomemory capacity of the different genotypes, seedlings were exposed to a severe HS of 44 °C for 90 min (triggering HS) a couple of days after the priming treatment.

### Generation of transgenic lines

To generate *HSFA2.OX* plants, the *HSFA2* open reading frame was amplified by PCR from Arabidopsis Col-0 leaf (heat-treated) cDNA, inserted into the pCR2.1 vector using the TA cloning kit (Invitrogen), and then cloned behind the cauliflower mosaic virus *35S* promoter previously inserted in the plant transformation vector pGreen0229 (Hellens *et al.*, 2000). To generate *HSFA2-IOE* plants, the *HSFA2* coding region was amplified by PCR from Arabidopsis Col-0 leaf cDNA, inserted into the plasmid pJET (Thermo Fisher Scientific), and then cloned via added *Xho*I and *Spe*I sites into the pER8 vector (Zuo *et al.*, 2000). Constructs were transformed into *Agrobacterium tumefaciens* strain GV3101. Transformation of Arabidopsis accession Col-0 with *Agrobacterium* was done using the floral dip method (Clough and Bent, 1998). Transformants were identified by selection on BASTA (*HSFA2.OX*) or hygromycin (*HSFA2-IOE*). Primer sequences are listed in Supplementary Table S1.

### Yeast one-hybrid assay

The yeast one-hybrid (Y1H) screen was done using a library of approximately 1400 Arabidopsis transcription factors representing an extended version of the library originally published by Castrillo *et al.* (2011), using a mating type protocol. The 500 bp *FtsH6* promoter upstream of the ATG start codon was amplified by PCR from Arabidopsis Col-0 genomic DNA and inserted upstream of the *HIS3* reporter in the plasmid pTUY1H via added *Xma*I and *Xba*I sites. The plasmid was transformed into yeast strain Y187, mating type α. Transcription factor library clones harboring the GAL4 activation domain in yeast strain YM4271 (mating type a) were mated with the yeast containing the *FtsH6* promoter (Castrillo *et al.*, 2011). Diploid cells were selected on SD/-Leu/-Trp/-His medium with different concentrations of 3-amino-1,2,4-triazol (3AT).

### Protein extraction and immunoblotting

Total protein was extracted from Arabidopsis seedlings using a phenol-based method as previously described (Sedaghatmehr *et al.*, 2016). Briefly, powdered tissue was homogenized in 0.7 M sucrose/0.5 M Tris/50 mM EDTA/0.1 M potassium chloride, pH 9.4, containing 2% (v/v) 2-mercaptoethanol and cOmplete^TM^ Protease Inhibitor Cocktail (Roche). The homogenate was mixed with an equal volume of phenol; after centrifugation in a microcentrifuge, proteins were precipitated from the phenol phase by adding 0.1 M ammonium acetate in methanol and overnight incubation at –20 °C. The protein pellet obtained after centrifugation was washed with methanolic ammonium acetate and then dissolved in 1% SDS. Protein concentration was determined using the BCA Protein Assay kit (Thermo Fisher Scientific). Proteins were resolved by 12% or 15% SDS-PAGE. PageRule Plus Prestained Protein Ladder (10–250 kDa; Thermo Scientific) was used as a protein molecular weight marker.

For immunoblot analysis, proteins were blotted via wet transfer on to a Protan nitrocellulose membrane (Sigma-Aldrich, 10401396). Rabbit anti-HSP21 polyclonal antibody (Abcam, ab80175; 1:1000) and rabbit anti-FtsH6 polyclonal antibody (Agrisera, AS05 094A; 1:1000) were used. IRDye 800CW-conjugated goat anti-rabbit IgG (H+L) antibody was used as secondary antibody at 1:10 000 dilution (LI-COR Biosciences). The Odyssey Infrared Imaging System (LI-COR Biosciences) was used for the detection of bands.

### RNA isolation and qRT–PCR

Total RNA from Arabidopsis seedlings was extracted using the RNeasy Plant Mini kit (Qiagen, Hilden, Germany). Synthesis of cDNA and quantitative reverse transcription–PCR (qRT–PCR) were performed as previously described (Balazadeh *et al.*, 2008). PCRs with SYBR GREEN as dsDNA-binding dye were run on an ABI PRISM 7900HT sequence detection system (Applied Biosystems Applera); *ACTIN2* and *GAPDH* were used as reference genes. Primer sequences were designed using QuantPrime (Arvidsson *et al.*, 2008; https://quantprime.mpimp-golm.mpg.de/) and are listed in Supplementary Table S1.

### Chromatin immunoprecipitation

For chromatin immunoprecipitation (ChIP) demonstrating the binding of HSFA2 to the *FtsH6* promoter, chromatin extract was isolated from primed and unprimed seedlings of *HSFA2prom:HSFA2-YFP*/*hsfa2* plants. The chromatin extract was isolated as described previously (Kaufmann *et al.*, 2010). Anti-GFP antibody beads (µMACS and MultiMACS GFP Isolation kits; Miltenyi Biotec) were used to immunoprecipitate protein–DNA complexes. After reversion of the cross-linking, DNA was purified by using a QIAquick PCR Purification Kit (Qiagen) and analysed by quantitative PCR (qPCR) using the primers listed in Supplementary Table S1. PCRs using SYBR GREEN as dsDNA-binding agent were performed using an ABI PRISM 7900HT sequence detection system.

### Arabidopsis gene codes

Gene codes for the genes used in this study, according to The Arabidopsis Information Resource (TAIR; https://www.arabidopsis.org/) are as follows: *ACTIN2*, *AT3G18780*; *APX2*, *AT3G09640*; *FtsH6*, *AT5G15250*; *GAPDH*, *AT1G13440*; *HSP21*, *AT4G27670*; *HSFA2*, *AT2G26150*.

## Results

### Yeast one-hybrid screen identifies HSFA2 as an *FtsH6*-binding transcription factor

**Fig. 1.**
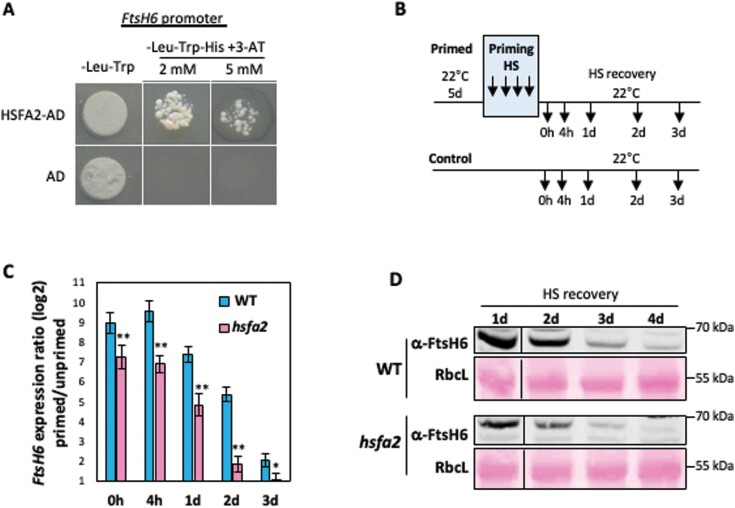
HSFA2 binds the *FtsH6* promoter and affects its transcription during the thermorecovery phase. (A) Binding of HSFA2 to the promoter of *FtsH6* (0.5 kb upstream of the *FtsH6* start codon) determined via Y1H assay. HSFA2-AD, HSFA2 fused to the yeast Gal4 activation domain; AD, Gal4 activation domain alone. (B) Priming protocol: 5-day-old WT and *hsfa2* plants were subjected to HS priming, a heat regime of 1.5 h at 37 °C, recovery for 1.5 h at 22 °C, and 45 min at 44 °C, after which plants were returned to normal growth conditions (thermorecovery phase). Arrows indicate harvest time points. (C) *FtsH6* expression in *hsfa2* mutant and WT seedlings after HS priming, at different time points in the thermorecovery phase, compared with unprimed controls. The y axis indicates the log_2_ fold ratio of gene expression between primed and unprimed samples. Data are the means ±SD of three biological replicates. Asterisks indicate statistically significant differences from WT (*P<0.05, **P<0.01; Student’s *t*-test). (D) Immunoblot analysis of FtsH6 protein in WT and *hsfa2* mutant seedlings during the thermorecovery phase (uncropped images of the immunoblots are shown in Supplementary Fig. S3). Seedlings were harvested for immunoblotting at days 1, 2, 3, and 4 after thermopriming. Immunodetection was performed using anti-FtsH6 antibody (α-FtsH6). RbcL, ribulose-1,5-bisphosphate carboxylase/oxygenase large subunit, Ponceau-stained (loading control; lower panels). The experiment was repeated with three biological replicates and similar results were obtained in all of them.

We previously reported that the expression of *FtsH6* is strongly induced by thermopriming and remains high during the thermorecovery/thermomemory phase (Sedaghatmehr *et al.*, 2016). We then intended to identify upstream transcriptional regulatory factors controlling the expression of *FtsH6.* To this end, we performed a Y1H screen using the 0.5 kb promoter fragment of the *FtsH6* gene (0.5 kb upstream of the *FtsH6* start codon) as a bait; screening of longer *FtsH6* promoter fragments in the Y1H system was not possible due to their strong self-activation property. In a screen with ~1400 Arabidopsis transcription factors we identified that HSFA2 activates the *HIS3* reporter in yeast (Fig. 1A). All HSFs harbor an N-terminal DNA-binding domain, which recognizes a conserved heat shock element (HSE) (5ʹ-nGAAn...TTCn3ʹ or 5ʹ-nTTCn…GAAn-3ʹ) in the promoters of HS-responsive genes (Nishizawa *et al.*, 2006; Scharf *et al.*, 2012). Accordingly, as shown below, HSFA2 binds to an *FtsH6* promoter region that contains multiple HSEs.

### HSFA2 positively regulates *FtsH6* expression during the thermorecovery phase

**Fig. 2.**
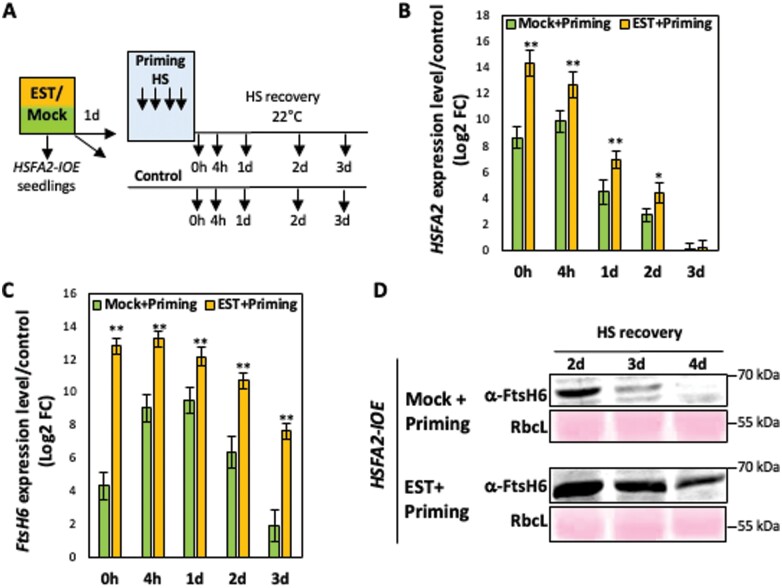
HSFA2 activates *FtsH6* transcription during the thermorecovery phase. (A) Five-day-old *HSFA2-IOE* seedlings were subjected to thermopriming, a heat regime of 1.5 h at 37 °C, recovery for 1.5 h at 22 °C, and 45 min at 44 °C, after which plants were returned to normal growth conditions (thermorecovery phase). (B) *HSFA2* expression is induced in *HSFA2-IOE* seedlings upon treatment with 12 μM estradiol (EST) compared with mock treatment (0.1% ethanol, v/v) during the thermorecovery phase. Seedlings were transferred to EST- or ethanol- (as control) containing MS medium 1 d before HS priming. (C) Induction of *HSFA2* expression by EST in *HSFA2-IOE* plants increases the expression of *FtsH6* at different time points (up to 3 d) into the thermorecovery phase, compared with primed mock-treated plants. Data are means ±SD of three biological replicates. FC, fold change. In (B) and (C), asterisks indicate statistically significant differences from the mock treatment (*P<0.05, **P<0.01; Student’s *t*-test). (D) Immunoblot analysis of FtsH6 protein in *HSFA2-IOE* seedlings during the thermorecovery phase (uncropped images of the immunoblots are shown in Supplementary Fig. S3). *HSFA2-IOE* seedlings were treated with EST or ethanol 1 d before thermopriming. The seedlings were harvested for immunoblotting after the thermopriming, at days 2, 3, and 4 into the thermorecovery phase. Immunodetection was performed using anti-FtsH6 antibody (α-FtsH6; upper panels). RbcL, ribulose-1,5-bisphosphate carboxylase/oxygenase large subunit, Ponceau-stained (loading control; lower panels). The experiment was repeated with three biological replicates and similar results were obtained in all of them.

To test whether HSFA2 is a genuine upstream regulator of *FtsH6*, we used an established thermomemory protocol (Fig. 1B) (Sedaghatmehr *et al.*, 2016) and examined the expression of *FtsH6* as well as *APX2*, a confirmed downstream target of HSFA2 (Nishizawa *et al.*, 2006), during the thermorecovery phase in seedlings of wild-type (WT) and *hsfa2* plants. By using qRT–PCR, we found that the induction of *FtsH6* expression was reduced (by more than ~2-fold) in *hsfa2* compared with WT at all examined time points of the thermorecovery phase (Fig. 1C). Similarly, the induction of *APX2* expression was decreased in *hsfa2* compared with WT during the thermorecovery phase (Supplementary Fig. S1A). Consistent with the reduced *FtsH6* transcript abundance in *hsfa2* plants, a lower FtsH6 protein accumulation (determined by western blot) compared with WT was evident during the thermorecovery phase (Fig. 1D). To validate the positive control of HSFA2 over *FtsH6*, we expressed *HSFA2* from an estradiol (EST)-inducible promoter (Zuo *et al.*, 2000) and tested the expression of *FtsH6* after EST induction. To this end, we transferred 4-day-old transgenic seedlings (*HSFA2-IOE*) to half-strength Murashige and Skoog (MS) medium containing 12 μM EST (or 0.1% ethanol in mock control experiments) and applied the priming stimulus 1 d later (Fig. 2A). qRT–PCR revealed enhanced expression of *HSFA2* in EST-treated *HSFA2-IOE* seedlings after priming up to 72 h into the recovery phase (Fig. 2B). Consequently, the induction of *FtsH6* and *APX2* expression was significantly greater in EST-treated *HSFA2-IOE* seedlings compared with ethanol-treated *HSFA2-IOE* seedlings during all time points of the thermorecovery phase (Fig. 2C; Supplementary Fig. S1B). In accordance with the elevated transcript level of *FtsH6* in EST-treated *HSFA2-IOE* seedlings, these plants accumulated more FtsH6 protein compared with mock-treated *HSFA2-IOE* seedlings at days 2–4 into the thermorecovery phase (Fig. 2D). Collectively, our data indicate that HSFA2 functions as a positive regulator of *FtsH6* expression after thermopriming and during the thermorecovery phase.

### HSFA2 activates *FtsH6* expression during thermorecovery by binding to its promoter

**Fig. 3.**
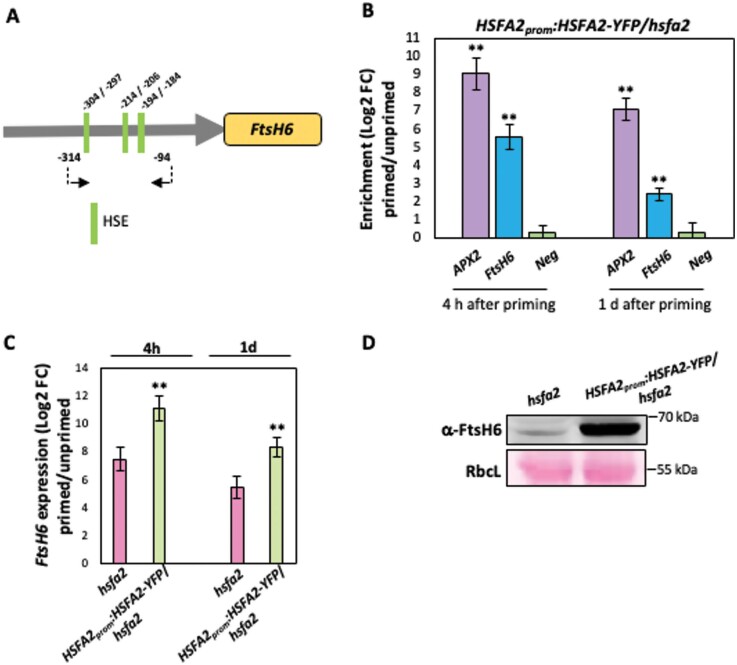
ChIP-qPCR demonstrates binding of HSFA2 to the *FtsH6* promoter during the thermorecovery phase. (A) qPCR primers, indicated by black arrows, annealing to the *FtsH6* promoter and flanking heat shock elements (HSE). (B) Seedlings of Arabidopsis plants expressing YFP-tagged HSFA2 under the control of the *HSFA2* promoter (*HSFA2*_*prom*_*:HSFA2-YFP*/*hsfa2*) were harvested after thermopriming at 4 h and 1 d into the thermorecovery phase, or under control conditions (unprimed), for the ChIP experiment. qPCR was used to quantify enrichment of the *FtsH6* promoter. As a positive control, primers annealing to promoter regions of an Arabidopsis gene containing an HSFA2 binding site (*APX2*) was used. As a negative control (Neg), primers annealing to the promoter regions of an Arabidopsis gene lacking an HSFA2 binding site (*CLAVATA1*; *CLV1*) were used. Data are means ±SD of three independent experiments. FC, fold change. Asterisks indicate statistically significant differences from the negative control (**P<0.01; Student’s *t*-test). (C) Transcript abundance of *FtsH6* measured by qRT–PCR in *HSFA2*_*prom*_*:HSFA2-YFP*/*hsfa2* and *hsfa2* seedlings at 4 h and 1 d into the thermorecovery phase, compared with unprimed plants. Asterisks indicate statistically significant differences from *hsfa2* (**P<0.01; Student’s *t*-test). (D) Immunoblot analysis showing FtsH6 protein in *HSFA2*_*prom*_*:HSFA2-YFP*/*hsfa2* and *hsfa2* seedlings at 1 d into the thermorecovery phase (α-FtsH6; an uncropped image of the immunoblot is shown in Supplementary Fig. S3). RbcL, ribulose-1,5-bisphosphate carboxylase/oxygenase large subunit, Ponceau-stained (loading control; lower panel). The experiment was repeated with three biological replicates and similar results were obtained in all of them.

To test whether HSFA2 directly regulates *FtsH6* expression *in planta*, we performed ChIP assays using transgenic Arabidopsis plants expressing an *HSFA2-YFP* (*YELLOW FLUORESCENT PROTEIN*) fusion from the native *HSFA2* promoter, in the *hsfa2* mutant (hereafter, *HSFA2*_*prom*_*:HSFA2-YFP*/*hsfa2*). Seedlings from *HSFA2*_*prom*_*:HSFA2-YFP*/*hsfa2* and *hsfa2* plants were harvested 4 h and 1d after thermopriming, and at the respective time points of control conditions (unprimed samples), for *FtsH6* expression analysis and ChIP-qPCR assays. ChIP-qPCR revealed enrichment of the *FtsH6* promoter harboring HSFA2 binding sites during the HS recovery phase (Fig. 3A, B). No enrichment was observed for a negative control fragment, that is, a promoter region of the *CLAVATA1* gene lacking an HSFA2 binding site, while enrichment was observed for the positive control gene *APX2* (Fig. 3B). In accordance with the binding of HSFA2 to the *FtsH6* promoter, we observed increased transcript levels and protein accumulation of FtsH6 in *HSFA2*_*prom*_*:HSFA2-YFP*/*hsfa2* seedlings compared with the *hsfa2* mutant during the thermorecovery phase (Fig. 3C, D). Our data thus demonstrate that HSFA2 is an upstream transcriptional regulator of *FtsH6* during thermorecovery.

### HSFA2 fine-tunes resetting of thermomemory by regulating *FtsH6* expression

**Fig. 4.**
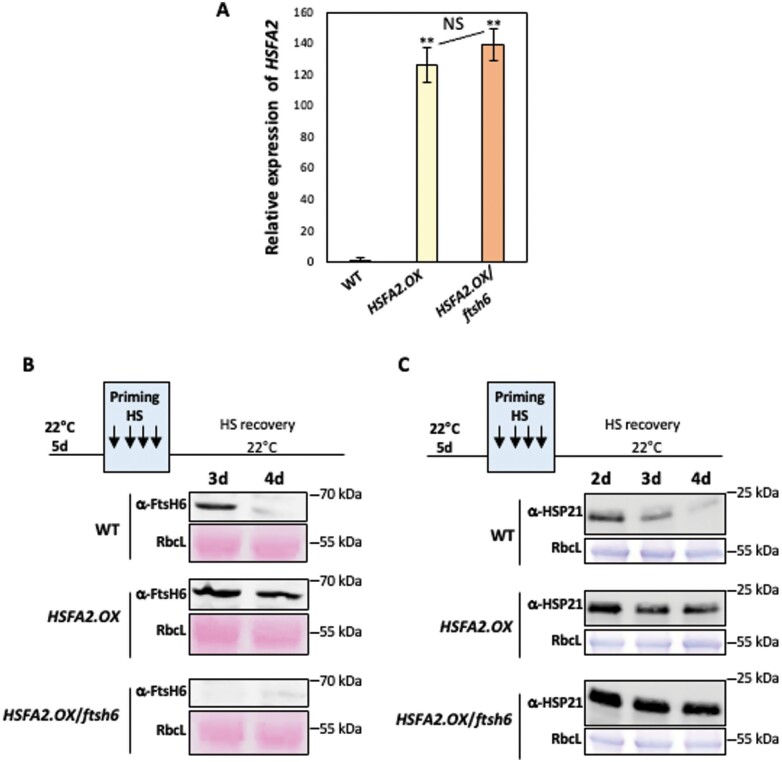
HSFA2 mediates the degradation of HSP21 through the induction of *FtsH6* expression during the recovery phase. (A) Elevated expression of *HSFA2* in 7-day-old seedlings of *HSFA2.OX* and *HSFA2.OX*/*ftsh6* plants grown under normal conditions compared with WT plants. Gene expression was analysed by qRT–PCR using the reference genes *ACTIN2* and *GAPDH.* Relative transcript levels are shown as 2^−ΔCt^ values. Asterisks indicate statistically significant differences from the WT (**P<0.01, Student’s *t*-test). NS, no significant difference between *HSFA2.OX* and *HSFA2.OX*/*ftsh6* plants. (B) Immunoblot analysis of FtsH6 protein in WT, *HSFA2.OX*, and *HSFA2.OX*/*ftsh6* seedlings during the thermorecovery phase. The seedlings were harvested for immunoblotting at days 3 and 4 into the thermorecovery phase. (C) Immunoblot analysis of HSP21 protein in WT, *HSFA2.OX*, and *HSFA2.OX*/*ftsh6* seedlings during the thermorecovery phase. The seedlings were harvested for immunoblotting at days 2, 3, and 4 into the thermorecovery phase. Immunodetection was performed using anti-FtsH6 (α-FtsH6) and anti-HSP21 (α-HSP21) antibodies, respectively. The lower panels in (B) and (C) show the accumulation of ribulose-1,5-bisphosphate carboxylase/oxygenase large subunit (RbcL) as a loading control; (B), Ponceau staining; (C), Coomassie Brilliant Blue staining. Uncropped images of the immunoblots in (B) and (C) are shown in Supplementary Fig. S3). The experiment was repeated with three biological replicates and similar results were obtained in all of them.

We previously reported that FtsH6 negatively regulates thermomemory by degrading plastidial HSP21 (Sedaghatmehr *et al.*, 2016). Considering the importance of HSFA2 for establishing thermomemory (Charng *et al.*, 2007; Lämke *et al.*, 2016) and its role in regulating *FtsH6* expression (Figs 1–3), we hypothesized that HSFA2 affects thermomemory differently in the presence or absence of FtsH6. To test this, we generated plants overexpressing *HSFA2* in both the Col-0 and *ftsh6* backgrounds (Fig. 4A). Consistent with an elevated expression of *HSFA2*, more FtsH6 protein accumulated in *HSFA2*-overexpressing plants (Col-0 background; *HSFA2.OX*) than in WT plants at days 3 and 4 into the recovery phase, while FtsH6 protein was not detected in *HSFA2.OX*/*ftsh6* plants, as expected (Fig. 4B). We then performed immunoblotting to determine the HSP21 protein accumulation at days 2–4 into the thermorecovery phase in transgenic plants overexpressing *HSFA2*. *HSFA2.OX* plants accumulated more HSP21 protein than WT plants at different time points into the recovery phase (Fig. 4C), in agreement with previous findings showing that HSFA2 positively regulates *HSP21* expression (Nishizawa *et al.*, 2006; Charng *et al.*, 2007). Importantly, the lack of functional FtsH6 in *HSFA2.OX*/*ftsh6* plants led to increased HSP21 accumulation compared with plants overexpressing *HSFA2* in the WT background (Fig. 4C), favoring the extension of thermomemory. Taken together, these findings suggest that HSFA2 mediates the resetting of HSP21 abundance to its pre-stress level via the activation of *FtsH6*.

### HSFA2 extends priming-induced thermotolerance particularly in the absence of FtsH6

**Fig. 5.**
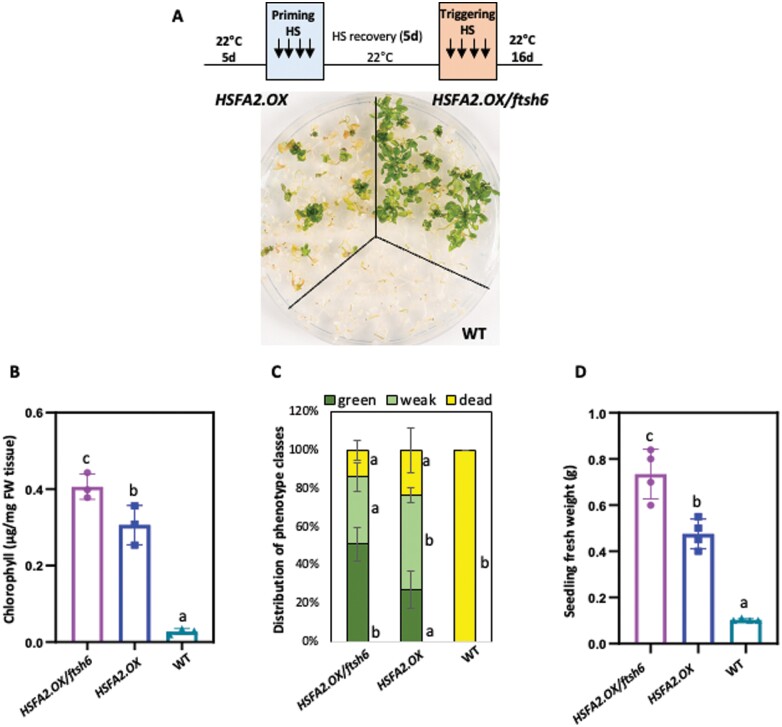
Overexpression of *HSFA2* enhances thermomemory capacity, especially in the absence of FtsH6. (A) Thermomemory of WT, *HSFA2.OX*, and *HSFA2.OX*/*ftsh6* plants. Seedlings were primed by moderate HS (1.5 h at 37 °C; 1.5 h of recovery at 22 °C; 45 min at 44 °C) and then returned to normal growth conditions for 5 d (thermorecovery phase). Seedlings were then exposed to a severe HS of 44 °C for 90 min (triggering HS) (shown schematically at the top). Following HS, seedlings were transferred to normal growth conditions and photographed after 16 d. (B–D) Quantification of results shown in (A): (B) chlorophyll content; (C) percentage of seedlings in different phenotype classes; (D) seedling fresh weight compared with control plants. Data are means ±SD (*n*=5 plates with ~25 seedlings each). Letters indicate significant differences among means (*P*<0.05; one-way ANOVA).

The crucial role of HSP21 for thermomemory (Sedaghatmehr *et al.*, 2016), together with our observation of higher accumulation of HSP21 in *HSFA2.OX*/*ftsh6* plants during the thermorecovery phase, prompted us to test whether the inactivation of *FtsH6* in the *HSFA2.OX* line affects HS priming. We used transgenic lines overexpressing *HSFA2* as well as WT plants to test their thermomemory behavior. In accordance with previous studies (Charng *et al.*, 2007; Friedrich *et al.*, 2021), *HSFA2.OX* plants survived significantly better after a post-memory HS (high-temperature stress applied 4 d or 5 d after the priming treatment) than the WT plants (Fig. 5A; Supplementary Fig. S2A). Interestingly, *ftsh6* plants overexpressing *HSFA2* performed considerably better than Col-0 plants overexpressing *HSFA2* during post-memory HS given 5 d after the priming treatment (Fig. 5A); a weaker effect was observed after a shorter (4 d) memory phase (Supplementary Fig. S2A). The *HSFA2.OX*/*ftsh6* plants showed more chlorophyll content (Fig. 5B; Supplementary Fig. S2B), a higher number of green seedlings (Fig. 5C; Supplementary Fig. S2C), and a higher seedling fresh weight (Fig. 5D; Supplementary Fig. S2D) than the *HSFA2.OX* plants. Collectively, our results demonstrate that HSFA2 directs the resetting of thermomemory through the induction of *FtsH6* expression during the thermorecovery phase.

## Discussion

Although diverse cellular mechanisms underlying the mounting of stress memory have been identified in recent years (Balazadeh, 2022), the molecular mechanisms controlling the forgetting of a past stress are less well understood. Priming affects the levels of many transcripts, proteins, and metabolites to enhance performance in stressful environments, but typically impairs maximal growth due to the energy and metabolic requirements associated with it (Crisp *et al*., 2016, 2017). Most of the stress-induced changes should, therefore, be reset to their pre-stress levels once the stress has abated, to allow redirection of nutrient and fixed carbon for regaining growth and production (Crisp *et al*., 2016, 2017). Readjustment and modification of stress-induced molecules during recovery after stress (memory/recovery phase) allow plants to reset their defense responses. Resetting defense mechanisms helps plants to focus on growth and reproduction (Hemme *et al.*, 2014).

Recently, we discovered that the plastidial small heat shock protein HSP21 is a functionally crucial component of thermomemory in Arabidopsis; variation in the HSP21 protein abundance contributes to differences in the thermomemory performance of Arabidopsis accessions (Sedaghatmehr *et al.*, 2016). Furthermore, we showed that a plastidial metalloprotease, FtsH6, is involved in HSP21 degradation, which resets the abundance of HSP21 to its pre-stress level during the recovery/memory phase (Sedaghatmehr *et al.*, 2016). In addition, autophagy, a major protein recycling pathway, contributes to resetting HSP21 abundance during the thermomemory phase (Sedaghatmehr *et al.*, 2021). Besides being involved in HSP21 removal, autophagy also contributes to resetting different classes of other HSPs in the later stages of the recovery phase (Sedaghatmehr *et al.*, 2019). Furthermore, the autophagy cargo receptors NEIGHBOR OF BRCA1 (NBR1) and ATG8-INTERACTING PROTEIN1 (ATI1) participate in selective degradation of memory components during HS recovery, thus helping plants to reset their memory of HS (Sedaghatmehr *et al.*, 2021; Thirumalaikumar *et al.*, 2021).

The expression of *HSP21* is up-regulated by HSFA2, a master transcriptional regulator of thermomemory in Arabidopsis (Charng *et al.*, 2007; Li *et al.*, 2010); in accordance with this, the *hsfa2* knockout mutant shows defective thermomemory. While *HSP21* transcripts are undetectable 24 h after thermopriming in *hsfa2* plants, they are highly abundant in WT plants up to 48 h after the priming stimulus (Charng *et al.*, 2007; Friedrich *et al.*, 2021). The expression of FtsH proteases is regulated in response to developmental and environmental cues. They are highly and specifically expressed in photosynthetic tissues, and their expression is strongly induced by high-light stress (Sinvany-Villalobo *et al.*, 2004; Wagner *et al.*, 2011; Wagner, 2012; Luo *et al.*, 2021). However, to our knowledge, direct upstream transcriptional regulators of *FtsH* genes have not been reported before. While the expression of *FtsH6* (and its orthologues) is typically low under non-stress conditions, expression is highly induced by heat treatment in diverse species including Arabidopsis (Sedaghatmehr *et al.*, 2016), *Brassica napus* (Yu *et al.*, 2014), wheat (Xue *et al.*, 2015), sorghum (Johnson *et al.*, 2014), and tomato (Sun *et al.*, 2006). Thus, the evolutionarily conserved heat induction of *FtsH6* expression suggests an important role of this metalloprotease in the response to HS. In this study we discovered that HSFA2 binds to the proximal 0.5 kb promoter of *FtsH6* and positively regulates its expression after priming and during the thermorecovery phase (Figs 1–3). In support of this, we show that thermopriming-induced *FtsH6* transcript and protein are reduced in the *hsfa2* mutant compared with WT (Fig. 1C, D), but enhanced in transgenic plants overexpressing *HSFA2* from a constitutive or a chemically inducible promoter (Figs 2, 4B). However, as the expression of *FtsH6* is not totally erased in the *hsfa2* mutant, additional transcription factors must be involved in controlling it. Although our Y1H screen did not identify transcription factors other than HSFA2 robustly activating the 0.5 kb *FtsH6* promoter, we cannot exclude the possibility that other transcription factors, including other HSFs, bind further upstream. Unfortunately, however, the strong autoactivation activity observed in yeast with longer *FtsH6* promoters (>0.5 kb) precluded screening for additional transcription factors binding to them. Future research needs to identify such upstream regulators and their wider control networks within physiological contexts, in both Arabidopsis and crop species, probably involving alternative approaches. Another important aspect requiring attention is precisely how FtsH6 protease affects thermomemory besides controlling the HSP21 protein abundance. FtsH6 might have several additional protein targets; identifying the ones controlling thermomemory will be a central task for future research.

**Fig. 6.**
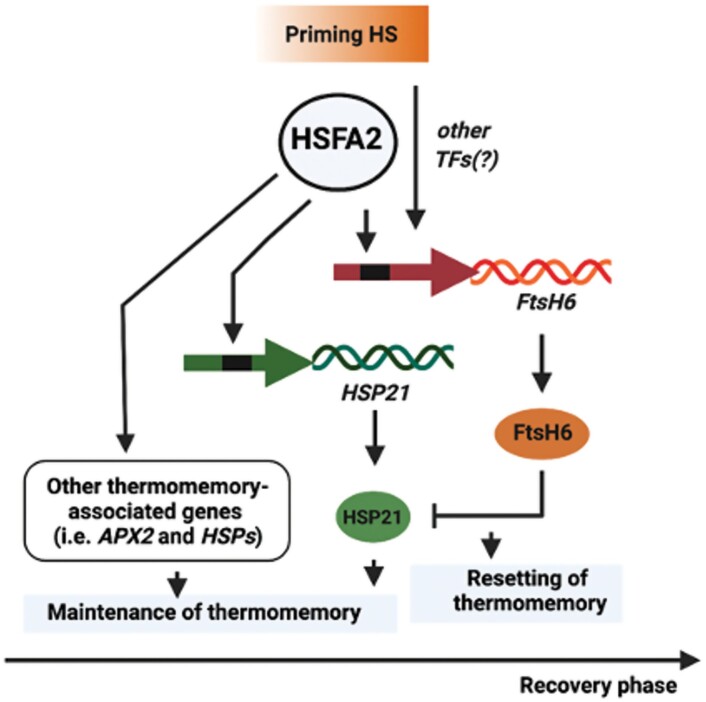
Model of the regulation of thermomemory/thermorecovery via HSFA2 and FtsH6. Thermopriming (moderate HS) induces the expression of *HSFA2*, a master regulator of thermomemory/thermorecovery. HSFA2 induces and maintains the expression of *HSP21* (and other thermomemory-associated genes), which is essential for the extension of thermomemory during the memory phase. HSFA2 also activates the expression of *FtsH6*, which is a component of resetting the memory of HS by targeting HSP21 for degradation. Consequently, HSFA2 creates a balance between prolongation and resetting of thermomemory. Figure created with BioRender.com.

Based on the data we have presented here, and considering previous results, we propose a model for the HSFA2–FtsH6 thermomemory module (Fig. 6). During thermopriming, HSFA2 activates the expression of both *HSP21* (Nishizawa *et al.*, 2006) and *FtsH6* (Figs 1–3), which are, respectively, positive and negative components of thermomemory. Upon progression into the thermorecovery/thermomemory phase, FtsH6 metalloprotease resets the HSP21 protein abundance to the pre-stress condition (Sedaghatmehr *et al.*, 2016); this restricts the duration of the thermomemory. A lack of functional FtsH6 in *HSFA2.OX*/*ftsh6* plants leads to a further increase in the abundance of HSP21 and thus enhanced thermomemory capacity compared with Col-0 plants overexpressing *HSFA2* (Figs 4, 5). Thus, HSFA2 fine-tunes the balance between prolongation and resetting of thermomemory affected by HSP21 and the resetting factor FtsH6 (Fig. 6). In consequence, HSFA2 functions as an important homeostasis control factor in plant thermomemory.

## Supplementary data

The following supplementary data are available at *JXB* online.

Fig. S1. HSFA2 regulates *APX2* transcription after a priming stimulus and during the thermorecovery phase.

Fig. S2. HSFA2 mediates resetting of thermomemory through the induction of *FtsH6* during the recovery phase.

Fig. S3. Uncropped images of immunoblots.

Table S1. Oligonucleotide sequences.

erac257_suppl_Supplementary_Material

## Data Availability

All data supporting the findings of this study are available within the paper and within its supplementary data published online.
