# Supplementary material for: Heat shock factor HSFA2 fine-tunes resetting of thermomemory via plastidic metalloprotease FtsH6
Source: J Exp Bot. 2022 Jun 15;73(18):6394–404. doi: 10.1093/jxb/erac257 (PMC9578354; doi:10.1093/jxb/erac257)
Supplement: erac257_suppl_Supplementary_Material [file erac257_suppl_supplementary_material.pdf]

Supplementary Figure S1

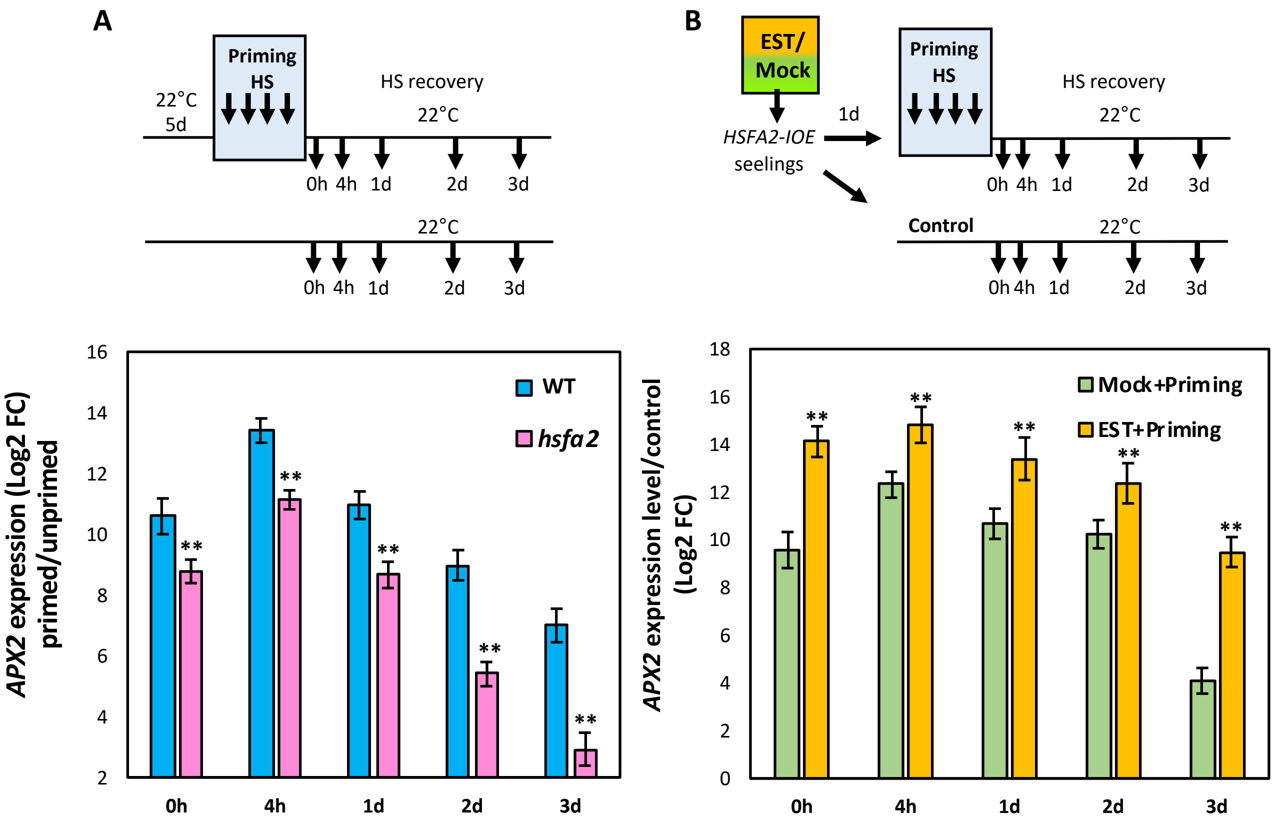

Supplementary Fig. S1. HSFA2 regulates *APX2* transcription after a priming stimulus and during the thermorecovery phase. (A) *APX2* expression in *hsfa2* and Col-0 seedlings after HS priming, at different time points into the thermorecovery phase, compared to unprimed controls. **(B)** *APX2* expression is induced in *HSFA2-IOE* seedlings upon treatment with 12 µM EST compared with mock treatment (0.1% ethanol) during the thermorecovery phase. As shown schematically, EST was added 1 day before HS priming to plates containing 0.5 MS medium. Induction of *HSFA2* expression by EST in *HSFA2-IOE* plants increases expression of *APX2* at 0, 4, 24, 48 and 72 h into the thermorecovery phase compared to primed, mock-treated plants. Data are the means of three biological replicates ± SD. FC, fold change. Asterisks indicate statistically significant difference from either WT (panel A) or mock treatment (panel B) ($P < 0.01$; Student's *t*-test).

Supplementary Figure S2

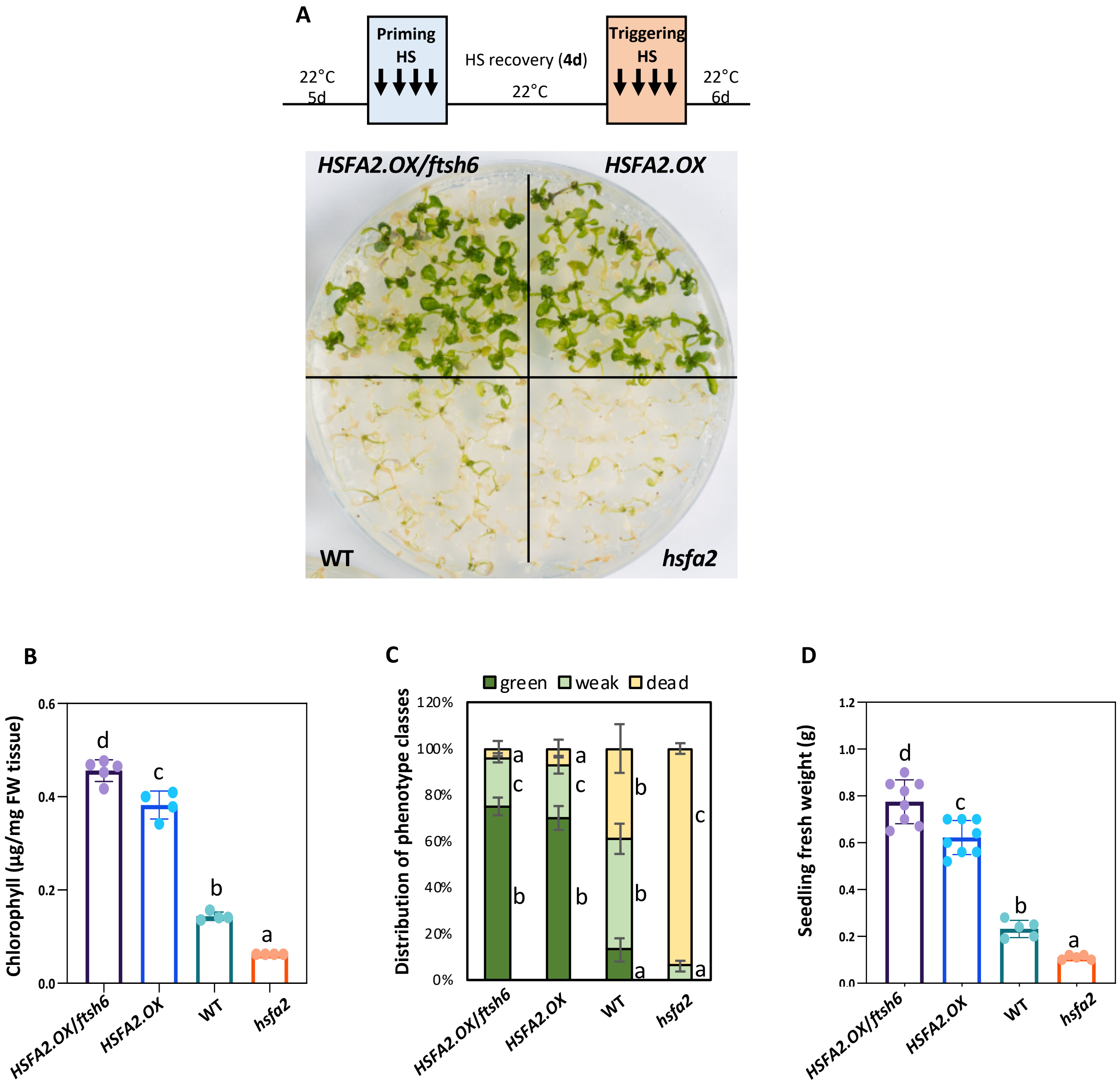

Supplementary Fig. S2. HSFA2 mediates resetting of thermomemory through the induction of *FtsH6* during the recovery phase. (A) The phenotype of the thermomemory of WT, *HSFA2.OX*, *HSFA2.OX/ftsh6*, and *hsfa2* plants. Seedlings were primed (1.5 h, 37°C; 1.5 h recovery at 22°C; 45 min, 44°C), and then returned to normal growth condition for 4 d (HS recovery phase). Seedlings were then exposed to a severe HS of 44°C for 90 min (triggering HS schematically shown on the top). Following HS, the seedlings were transferred to normal growth condition and photographed after 6 days. **(B)**, **(C)** and **(D)** Quantification of results shown in panel A. **(B)** Chlorophyll content. **(C)** The percentage of seedlings in different phenotype classes. **(D)** Seedling fresh weight compared to control plants. Means ± SD are given (n = 5 plates with ~25 seedlings each). Letters indicate significant differences among means ($P < 0.05$; one-way ANOVA).

Supplementary Figure S3

Figure 1D

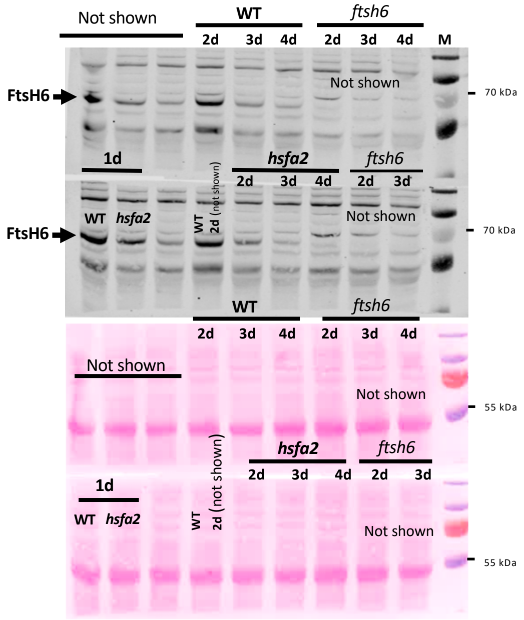

Figure 4B

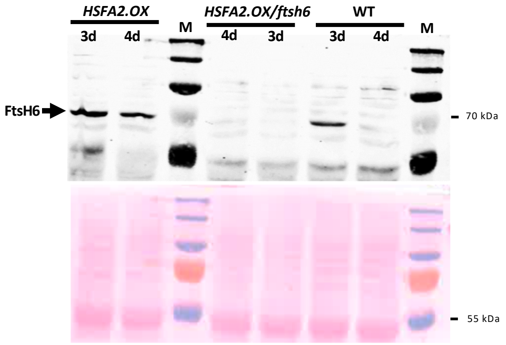

Figure 2D

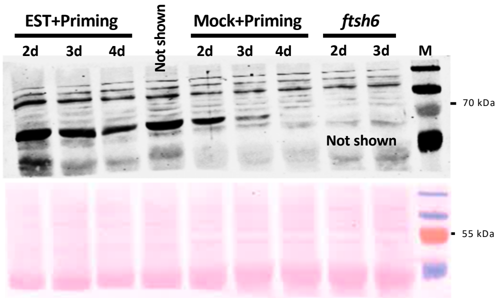

Figure 3D

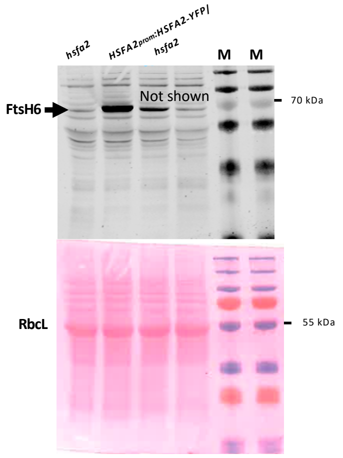

Figure 4C

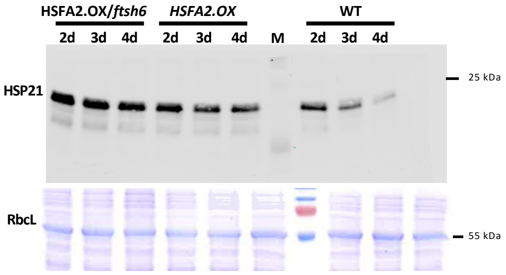

Supplementary Fig. S3. Uncropped images of immunoblots. M, molecular weight marker.

Supplementary Table S1. Oligonucleotide sequences.

Primers for qRT-PCR

Gene name	Gene ID	Primer Fwd sequence (5´-3´)	Primer Rev sequence (5´-3´)
HSFA2	*AT2G26150*	TCGTCAGCTCAATACTTATGGATTC	CACATGACATCCCAGATCCTTGC
FtsH6	*AT5G15250*	GCCGGAATGGAAGGGACAAAGATG	ATCATGACCCTCCGTCAAAGTCG
APX2	*AT3G09640*	ATTGTCTGGTGGACACACCTTGGG	TTGGTGTCCATGCACCCTCGAATC
ACTIN2	*AT3G18780*	TCCCTCAGCACATTCCAGCAGAT	AACGATTCCTGGACCTGCCTCATC
GAPDH	*At1G13440*	TTGGTGACAACAGGTCAAGCA	AAACTTGTCGCTCAATGCAATC

Constructs

Construct name	Forward primer sequence (5'-3')	Reverse primer sequence (5'-3')	Sites added
HSFA2.IOE	CTCGAGATGGAAGAACTGAAAGTGGAAATGG	ACTAGTTTAAGGTTCCGAACCAAGAAAACC	added *Spe*I and *Xho*I cloning site underlined
HSFA2.OX	CCCGTTTAAACATGGAAGAACTGAAAGTGGAAATGG	CCCTTAATTAATTAAGGTTCCGAACCAAGAAAACC	added *Pme*I and *Pac*I cloning site underlined
FtsH6 prom (Y1H)	CCCGGGATGTTAGAATTGTTATGTCTAGTGAGG	TCTAGATTTCATCAAAGTTAAGATATTTGTTCT	added *Xma*I and *Xba*I cloning site underlined

Primers for ChIP-qPCR

Gene name	Primer Fwd sequence (5´-3´)	Primer Rev sequence (5´-3´)
APX2	ACTAGCACTTTCTTCTGTGATCG	TTCCGGGTAACTCTTCTTCACC
FtsH6	GGAGAGTGTAGAAATTCGAAGGTTG	TCGATGGGTGTGTACAAGATTG
CLAVATA1	ACGGTCAGATCAACGCCG	TCTGCTGATCTTGCAGGTAG
